# Supplementary material for: MetaRibo-Seq measures translation in microbiomes
Source: Nat Commun. 2020 Jun 29;11:3268. doi: 10.1038/s41467-020-17081-z (PMC7324362; doi:10.1038/s41467-020-17081-z)
Supplement: Supplementary file 10 — Supplementary Data 7 [file 41467_2020_17081_MOESM10_ESM.zip › File2/Confidence_High_Taxonomy/24702_out.krona.html]

Javascript must be enabled to view this page.

members
magnitude
magnitudeUnassigned
count
unassigned
taxon
rank

24702\_out

3

3
superkingdom
2

phylum
1224
1

28211
class
1

SRS146812\_contig\_number\_60358

2
976
phylum

2
200643
class

2
order
171549

171550
family
1

1
genus
239759

1

SRS015694\_contig\_number\_2845
1118061
species

171552
family

SRS144135\_contig\_number\_contig-100\_2971.100360
1
